# Effectiveness of simulation-based training for manual small incision cataract surgery among novice surgeons: a randomized controlled trial

**DOI:** 10.1038/s41598-021-90410-4

**Published:** 2021-05-26

**Authors:** Akshay Gopinathan Nair, Chetan Ahiwalay, Ashish E. Bacchav, Tejas Sheth, Van Charles Lansingh, S. Swaroop Vedula, Venudhar Bhatt, Jagadesh C. Reddy, Pravin K. Vadavalli, Smita Praveen, Nikhilesh Anil Wairagade, Jeff Pettey

**Affiliations:** 1 HelpMeSee Inc., 703 A, Supreme Business Park, Powai, Hiranandani, Mumbai, 400 076 India; 2 HelpMeSee Inc., New York, USA; 3 L V Prasad Eye Institute, Banjara Hills, Hyderabad, India; 4 Sankara Nethralaya, Nungambakkam, Chennai, India; 5 Mahatme Eye Bank and Eye Hospital, Somalwada, Nagpur, India; 6 John A. Moran Eye Center, Salt Lake City, UT USA

**Keywords:** Outcomes research, Lens diseases

## Abstract

This study was designed to determine the effect of a novel simulation-based training curriculum for scleral tunnel construction in manual small incision cataract surgery (MSICS) compared with traditional training. In this multicenter, investigator-masked, randomized clinical trial, resident surgeons within 3 months of matriculation with minimal or no prior experience with MSICS were assigned either to simulation-based training, the Experimental Group (EG), or to conventional training, the Control Group (CG). EG residents were trained to perform scleral tunnel construction using a simulation-based curriculum (HelpMeSee Eye Surgery Simulator), while residents in the CG followed institution-specific curriculum before progressing to live surgery. Surgical videos of the first 20 attempts at tunnel construction were reviewed by masked video raters. The primary outcome was the total number of any of 9 pre-specified errors. On average, the total number of errors was 9.25 (95% CI 0–18.95) in the EG and 17.56 (95% CI 6.63–28.49) in the CG (*P* = 0.05); the number of major errors was 4.86 (95% CI 0.13–9.59) in the EG and 10.09 (95% CI 4.76–15.41) in the CG (*P* = 0.02); and the number of minor errors was 4.39 (95% CI 0–9.75) in the EG and 7.47 (95% CI 1.43–13.51) in the CG (*P* = 0.16). These results support that novice surgeons trained using the novel simulation-based curriculum performed fewer errors in their first 20 attempts at tunnel construction compared to those trained with a conventional curriculum.

## Introduction

Surgical expertise is acquired through deliberate practice; in other words, surgical experts are made, not born^1^. Improvement in surgical skill is associated with better outcomes and fewer surgical complications, especially in cataract surgery^2^. In the traditional training model in ophthalmology, trainees are assumed to be competent upon completing a certain number of surgical procedures under supervision^3^. The traditional training model is largely reliant upon access to experienced supervisors/educators and adequate teaching opportunities in the operating room. However, teaching in the operating room is limited by the competing priorities of optimizing quality and cost of patient care. Furthermore, surgery performed by trainees is associated with increased complication rates and poor patient outcomes^4–8^. Thus, it can be argued that the traditional training model is not sustainable, particularly in settings where training skillful surgeons is critical for public health, e.g., through widespread access to safe and effective cataract surgical services.

Simulation can potentially minimize trainees’ learning curves in the operating room by enabling deliberate practice in a low-risk environment. Simulation also offers the opportunity to receive feedback in the form of objective measures of performance, and it presents a wide range of clinical scenarios to trainees without putting patients at risk^8,9^. Numerous methods have been used as alternatives and integrated into surgical training, including artificial eyes, cadaver eyes, animal eyes and various virtual reality simulators are being integrated into surgical training^10^, but many VR simulators are limited in their fidelity, realism and effectiveness with regard to surgeons’ learning curves.

MSICS remains one of the most commonly performed surgeries worldwide^11^. The efficient utilisation of MSICS at high-volume surgical centres has been shown to be effective in delivering high quality outcomes^12^. Therefore, training ophthalmologists to perform the MSICS procedure safely and efficiently is of major ophthalmic public health significance^13^. At present, there is very little on efficacy of simulation-based surgical education for the SICS technique^14^.

Most VR simulators for cataract surgery have been developed for phacoemulsification^15^, including the Eyesi simulator, MicroVisTouch, PhacoVision, and the Phantom Phaco simulator, among others^16–20^. The training curricula developed using these VR simulators are heterogeneous across studies, with little evidence of reproducibility^3^. Furthermore, evidence on effectiveness of these VR simulators is limited to a few randomized controlled trials (RCTs) that did not evaluate the effect of training with a VR simulator-based curriculum on intraoperative performance beyond the first procedure^21^. Finally, to our knowledge, the effectiveness of VR simulation-based training curricula for manual small incision cataract surgery (MSICS) has yet to be studied. MSICS is the recommended alternative technique to replace phacoemulsification when the requisite equipment and expertise are not available. Surgeons with skill in MSICS are essential to address the global backlog in cataract surgical services^22,23^. High fidelity VR simulators have numerous advantages over task trainers, synthetic models and animal tissue models. Ethical concerns have arisen over the use of animals as surgical simulators. On the other hand, modern day VR simulators offer high-fidelity and anatomically correct simulations that are entirely reusable. VR surgical simulators offer a direct advantage over other simulators by letting trainees practice repeatedly, without supervision, while receiving direct feedback from the simulator itself^24^.

**Figure 1:**
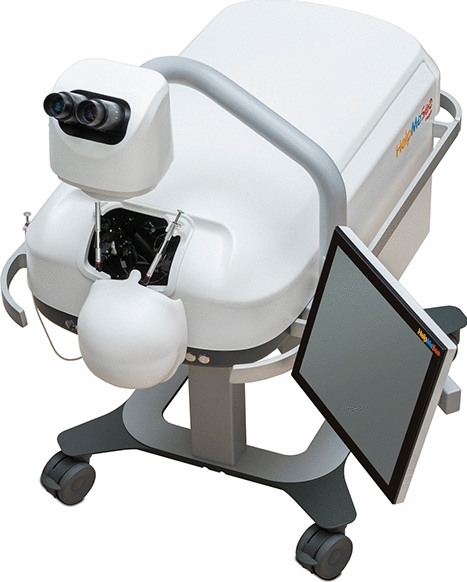
The HelpMeSee Eye Surgery Simulator (The authors have the permission from HelpMeSee Inc. to use this figure).

**Figure 2:**
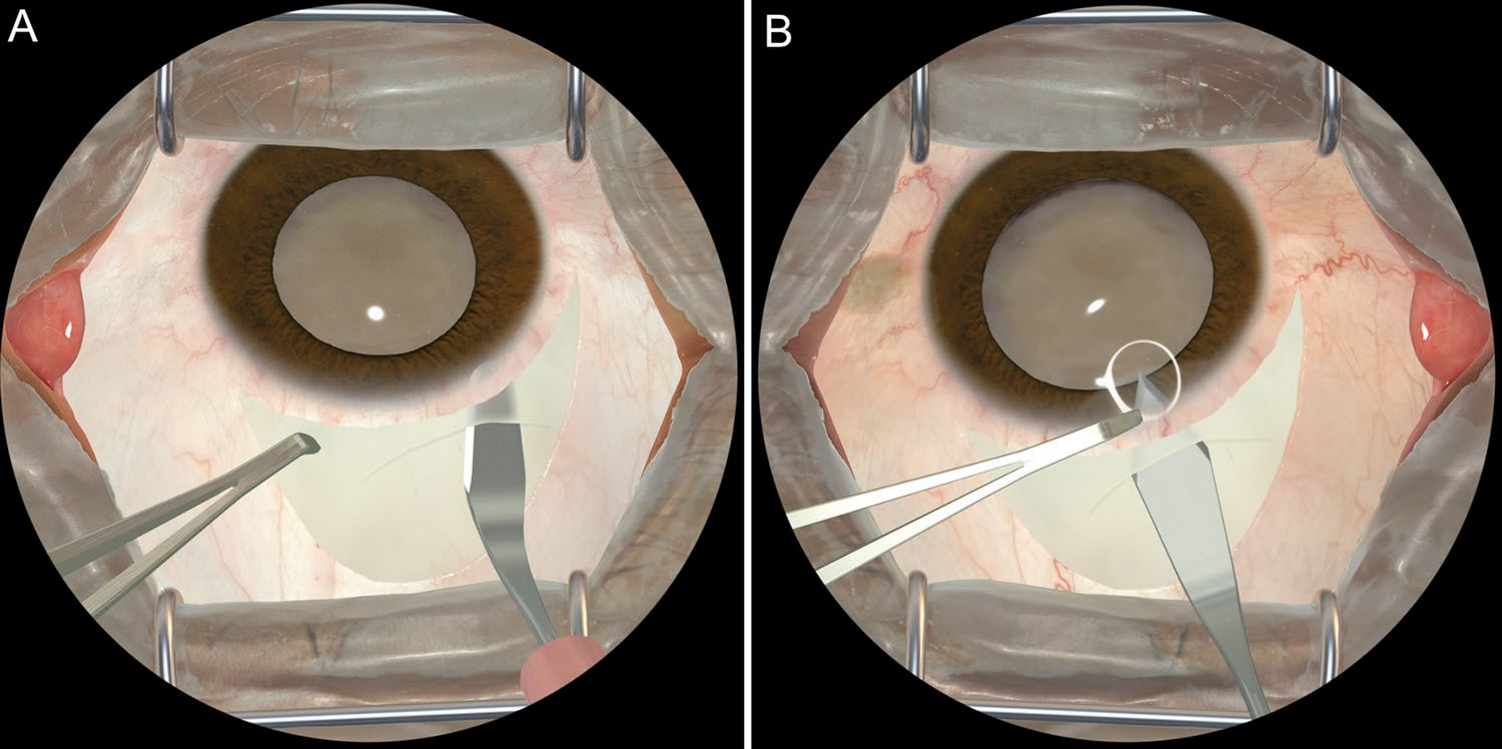
Surgeon’s view of the simulated surgery in the HelpMeSee Eye Surgery Simulator. (**A**) Depicts tunnel dissection with a crescent knife. (**B**) A keratome being used to enter the eye through the tunnel.

The HelpMeSee Eye Surgery Simulator is a VR simulator developed by HelpMeSee, Inc., New York, USA to support training in MSICS (Fig. 1). The simulator combines high quality computer graphics integrated with a physics model of surgical activities in MSICS and the ability to provide tactile feedback (Fig. 2). The HelpMeSee Eye Surgery Simulator is designed to provide a near-realistic experience of the activities during MSICS which are necessary for surgeons to effectively acquire skills. Our objective in this study was to determine the effectiveness of the novel HelpMeSee Eye Surgery simulator-based curriculum for scleral tunnel construction on intraoperative performance of surgical trainees early in their learning curve, i.e., the first 20 MSICS procedures they attempted in the operating room.

## Methods

This study was an early phase multi-center RCT approved by the Institutional Review Boards at all three participating institutes: Hyderabad Eye Research Foundation, LV Prasad Eye Institute Ethics Committee; Vision Research Foundation Ethics Committee, Sankara Nethralaya and the Institutional Ethics Committee, Mahatme Eye Bank & Eye Hospital. (ClinicalTrials.gov Identifier: NCT04450420; Date of first registration: 29/06/2020). Participants for this study were ophthalmology residents with minimal or no surgical skills in MSICS, recruited from five sites in three tertiary care academic institutions in India: LV Prasad Eye Institute (Hyderabad, Visakhapatnam, and Bhubaneshwar), Mahatme Eye Hospital (Nagpur), and Sankara Nethralaya (Chennai). Participants were recruited between September 1, 2018 and November 30, 2018 after obtaining informed consent. The study was conducted in accordance with the ethical standards of the institutional ethics committee and adhered to the Declaration of Helsinki, 1964 and its later amendments or comparable ethical standards. This study conforms to CONSORT 2010 guidelines and a CONSORT checklist has been provided.

Residents who met the following inclusion criteria were eligible to participate in this study: (1) enrolled in a training program at a participating institution; (2) within 3 months of matriculation; (3) self-reported prior experience with fewer than 20 MSICS procedures as the primary surgeon and no more than 5 procedures within the six months preceding enrollment in this study. Participants who met any of the following criteria were excluded: prior experience with the HelpMeSee simulator or participation in the HelpMeSee simulation-based training curriculum.

Eligible participants who consented were assigned to either the simulation-based curriculum (EG for experimental group) or traditional training (CG for control group). Assignment was stratified by site and by whether trainees reported performing tunnel construction in fewer than 5 procedures in the 6 months preceding enrollment into the study. A methodologist who was not involved in participant recruitment generated a blocked random number sequence (fixed block size of 2) using the randomizeR package in R. Assignments were provided to the study investigators after participants were evaluated for eligibility and enrolled into the study.

The intervention in EG was a structured, reproducible training curriculum for scleral tunnel construction based on the high-fidelity HelpMe See Eye Surgery Simulator. This curriculum included self-study using an eBook developed by HelpMeSee, instructor-led classroom instruction, hands-on dry-lab activities, training with the HelpMeSee Eye Surgery Simulator, and a live surgery phase. The simulation-based training curriculum for trainees in the EG spanned 6 days (35 h total), of which 80% of the time was dedicated to hands-on training on the simulator. Trainees were allowed time off from their clinical responsibilities in order to attend the simulation-based training phase. During the live surgical training phase for the EG, a surgeon from HelpMeSee was physically present in the operating room along with the supervising surgeon from the participating institution for the first 20 procedures in which they attempted tunnel construction. The HMS surgeon was present in the operating room solely as an observer during the live surgical training phase.

In the CG, trainees participated in the conventional curriculum specified by their institution without any additional input from HelpMeSee surgeons. Our goal was to evaluate the effectiveness of the novel curriculum; therefore, training in CG followed the standards at each participating institution. Participants in the CG undertook live surgical training under the supervision of mentor surgeons from the participating institutions.

The primary outcome was the total number of incident errors during the first 20 procedures in which trainees performed tunnel construction. Pre-specified errors targeted for this study were classified as major (uveal prolapse, buttonhole incision, premature entry, Descemet membrane detachment, and laceration of lateral walls or roof of tunnel), and minor (corneal endothelial touch, contact with iris, and contact with the lens). The total numbers of major and minor incident errors during the first 20 procedures in which trainees performed tunnel construction were analyzed separately as secondary outcomes.

Video review was the pre-specified mode of ascertaining outcomes. The videos were captured and stripped of identifiers by each participating institution. Each video was viewed and assessed by a set of three ophthalmologists who were experienced surgeons. These were randomly selected from a pool of 10 selected video reviewers. Each video reviewer was instructed in assessment of the study-specific errors. They were required to evaluate a set of calibration videos to detect any substantial deviations in their ratings over time. Videos grouped in a set of ten cases were prepared and assigned to the video raters. The set contained randomized and masked videos from both the groups, and the order of the surgical cases was randomized. Video raters were not informed about the group assignment or given any identifier about the trainee being evaluated. Data from the video reviews collected on paper forms was manually entered into a database with independent verification in a random 10% sample. The majority assessment from the three video raters was used for analysis.

Analysis was by intention to treat. Trainees were analyzed in the groups to which they were assigned. Assuming 40 errors on average in the first 100 procedures in which trainees perform tunnel construction and a standard deviation of 8, a study including 18 trainees was expected to have at least 80% power to detect an effect size of 1.5 or larger. Missing data in video ratings because of failure to record video for reasons not related to random assignment were imputed using intraoperative ratings.

A generalized linear model was fitted to include the group effect, prior experience with tunnel construction in the preceding 6 months, their interaction, and the study site. An F-test from type II sums of squares in an analysis of variance was the pre-specified measure of statistical significance of the group effect. Estimates are reported along with 95% confidence intervals (CIs); the level of significance was 0.05. No subgroup analyses were pre-specified in the protocol. All analyses were performed using R (version 3.6.1).

## Results

**Figure 3:**
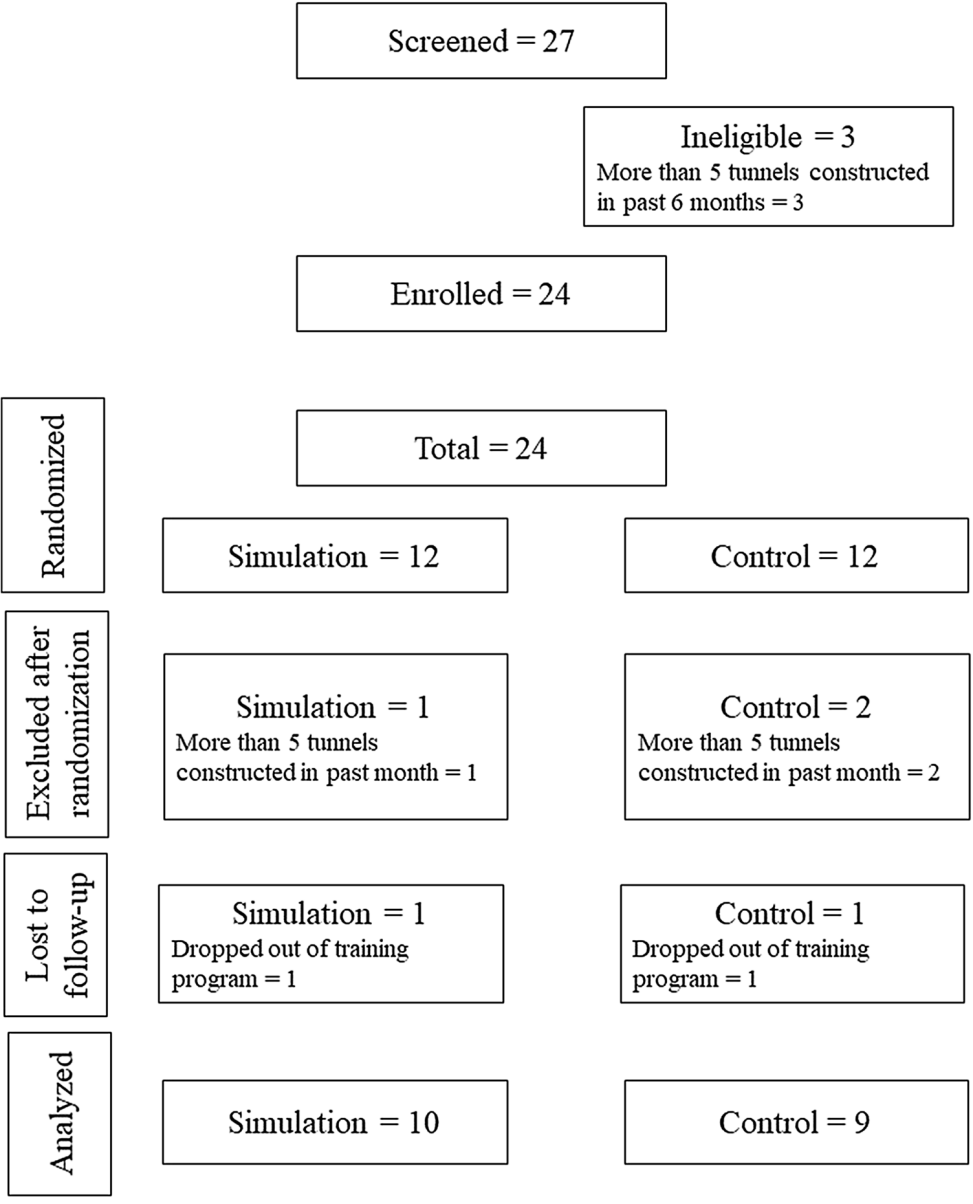
CONSORT flowchart for the study.

The CONSORT flowchart is shown in Fig. 3. A total of 24 trainees were randomized, including 10 from LV Prasad Eye Institute, 5 from Mahatme Eye Hospital, and 9 from Sankara Nethralaya. Three trainees were excluded after randomisation because of factors beyond the control of study investigators that led to trainees no longer meeting eligibility criteria. In addition, one trainee in each group dropped out of the training program, but there was no reason to believe that this was associated with study participation. Thus, 10 trainees in the EG and 9 in the CG were analyzed. One trainee in each group participated in the other group and they were analyzed as assigned.

Table 1 shows characteristics of trainees. The mean duration between the 1st and 20th surgeries was 25 days (SD 28.2 days) in the EG and 51.4 days (SD 28.7 days) in the CG. The mean interval between successive procedures during live surgery phase was 1.0 day for the EG (SD 1.48 days) and 2.0 days for the CG (SD 1.5 days).

**Table 1:** Characteristics of trainees as assigned and analysed in this study.

Variable	Control (N = 9)	Simulation (N = 10)
**Age; N (%)**		
25–30 years	8 (89)	7 (70)
31–35 years	1 (11)	3 (30)
**Sex; N (%)**		
Female	7 (78)	7 (70)
Male	2 (22)	3 (30)
**Reported handedness; N (%)**		
Right	8 (89)	10 (100)
Left	1 (11)	0 (0)
**Year of training; N (%)**		
PGY-1	7 (78)	5 (50)
PGY-2	1 (11)	1 (10)
PGY-3	0 (0)	1 (10)
Fellow	1 (11)	3 (30)
**Prior degree for fellows; N (%)**		
MS	0 (0)	1 (33.33)
DO	0 (0)	1 (33.33)
DNB	1 (100)	1 (33.33)
**Procedures performed as lead surgeon; Mean (SD)**		
Phacoemulsification	0 (0)	0.43 (1.13)
MSICS	6.67 (11.18)	16.30 (22.24)
ECCE	1 (2.65)	1 (1.73)
MSICS procedures performed as first assistant; Mean (SD)	3 (5.74)	18.78 (33)
**MSICS procedures in which participant constructed tunnel; Mean (SD)**		
Total	5.22 (10.02)	7.30 (11.36)
Within past 6 months	1.89 (3.82)	2.40 (3.89)
**Trainees in categories by tunnel construction reported in past 6 months**		
Less than 5	7	7
5 or more	2	3
**Trainees reporting at least 1 complication during MSICS in past 6 months; N (%)**		
Laceration	1 (11)	3 (30)
Button hole	0 (0)	0 (0)
Premature entry	0 (0)	0 (0)
**Prior training in simulation; N (%)**		
Virtual reality	1 (11)	2 (20)
Wet lab	8 (89)	7 (70)
**Number of hours trained in simulation; Mean (SD)**		
Virtual reality	NA (NA)	1 (NA)
Wet lab	4.33 (3.83)	12.50 (4.32)

*PGY* post-graduate year, *MS* Master of Surgery, *DO* Diploma in Ophthalmology, *DNB* Diplomate of the National Board, *MSICS* manual small incision cataract surgery, *ECCE* extra-capsular cataract extraction, *SD* standard deviation, *NA* not applicable.

**Figure 4:**
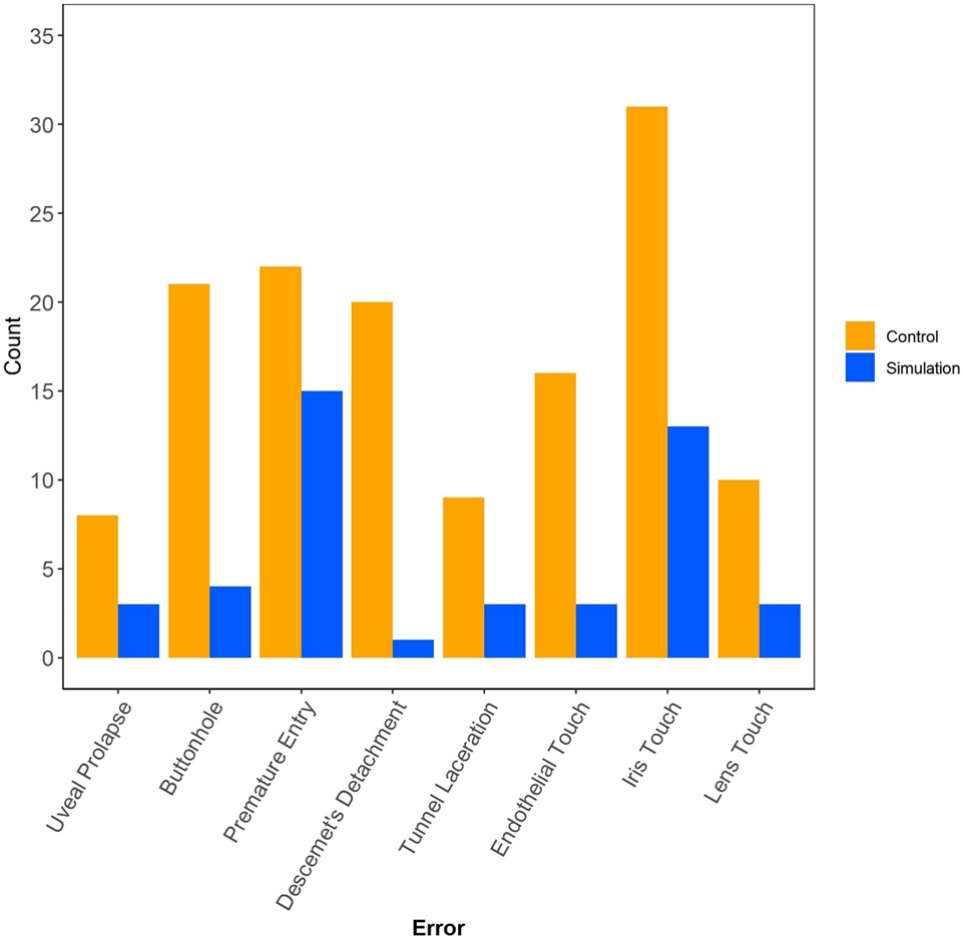
Counts of individual errors observed in the initial 20 procedures performed by TCC-PASTE participants.

**Figure 5:**
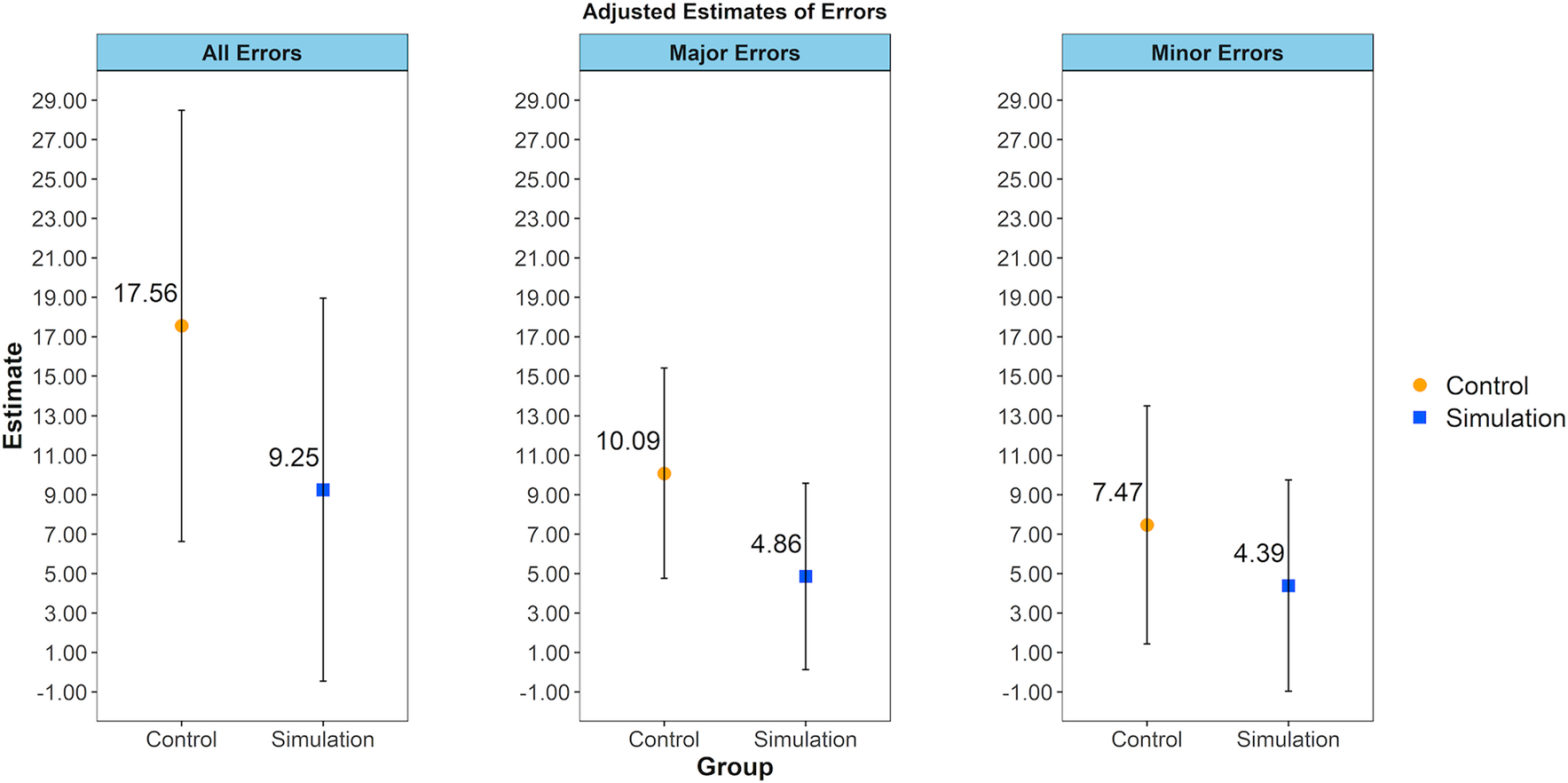
Adjusted estimates for average counts of errors over the initial 20 procedures performed by trainees in TCC-PASTE.

Figure 4 shows the unadjusted total counts for errors observed in the EG and the CG across 380 procedures. Errors were uniformly more frequent in the CG than in the EG. Figure 5 shows estimates from adjusted analyses. On average, over the initial 20 procedures performed by trainees in TCC-PASTE, the total number of errors was 17.56 (95% CI 6.63–28.49) in the control group and 9.25 (95% CI 0–18.95) in the simulation group; the number of major errors was 10.09 (95% CI 4.76–15.41) in the control group and 4.86 (0.13–9.59) in the simulation group; and the number of minor errors was 7.47 (95% CI 1.43–13.51) in the control group and 4.39 (95% CI 0–9.75) in the simulation group. The effect of the group variable was not statistically significant for total and minor errors (*P* = 0.05 and 0.16, respectively), and statistically significant for major errors (*P* = 0.02). The observed effect translates to a 47% reduction in total number of errors, 52% reduction in major errors, and 41% reduction in minor errors with the VR simulation-based curriculum among trainees early in their learning curve.

## Discussion

Our findings show that a VR simulation-based training curriculum is more effective than conventional training. The novel curriculum reduced by nearly one-half the incident error in the operating room among trainees performing tunnel construction early in their learning curve. This is a meaningful effect with considerable potential impact on patient safety in the operating room. This is also the first RCT evaluating effectiveness of a VR simulation-based training curriculum for MSICS, based on a recent systematic review^25^. Previous studies evaluating VR simulation-based training were either uncontrolled, non-randomized, or based on audits of clinical databases.

The magnitude of effect observed in our study is consistent with that found by other studies in trainees early in their learning curve. Using a synthetic eye model for simulation of the entire MSICS procedure, Dean, et al., reported a 70% reduction in posterior capsule rupture in procedures performed by residents in their first year of training^14^. Unlike the previous study, our RCT evaluated a greater number of major and minor errors during tunnel construction.

The observed effect on errors in the operating room is best explained as a direct consequence of training on the simulator. Face and content validity of the HelpMeSee Eye Surgery Simulator for tunnel construction has been established^26^. Trainees in EG were able to repeatedly practice each step in tunnel construction, including scleral groove creation, lamellar scleral tunnel dissection, paracentesis, viscoelastic injection and keratome entry until they were assessed by instructors as competent in individual task performance. Such repetitive deliberate practice is possible with no additional cost in VR simulation unlike simulation with synthetic or animal eyes. Trainees performed these tasks in sequence as a continuous procedural performance. The trainees were thus able to understand the effect of each of these steps on the subsequent one, as well as the impact that an error in one step can have on the subsequent tasks. Trainees in the CG undertook the standard training program as set by their institutions, which in most cases included some form of wet-lab training before they progressed to live surgical training in the operating room. Thus, the likely explanation for the observed effect favouring EG is the guided deliberate practice on the HelpMeSee Eye Surgery Simulator.

Our study findings may not be attributed to confounding or selection bias. Prior experience of participating trainees is not a likely explanation of the observed findings. Despite a slightly higher number of trainees in the EG, there was no significant difference in the number of trainees who reported having performed scleral tunnel construction in 5 or more procedures in the 6 months preceding enrollment. However, participants in the EG reported having more wet-lab training than those in the CG. The impact of prior wet-lab training on acquiring skill in VR simulation and its subsequent transfer to the operating room is not well known. Our study was not designed to estimate this interaction effect. In addition, differential assessment of outcomes is not a likely explanation of the observed effect with the HelpMeSee Eye Surgery Simulator-based training curriculum. Videos were rated by expert surgeons who were blinded to the identity of the trainees, group assignment, and the sequence of the procedures. Finally, the training curriculum differed between EG and CG by design. Our goal was to estimate effectiveness of the curriculum in EG compared with that in CG; therefore, trainees in CG received the current standard curriculum at their institution. While some suggest this design may lead to ethical concerns^25^, These are unfounded because there is still insufficient evidence of the effect of VR simulation-based training to influence equipoise. Besides, randomization and other methodological safeguards are critical to obtain unbiased estimates of effect.

There are two major barriers to addressing the pressing need for cataract surgical services across the globe: cost and the availability of skilled surgeons^27^. Despite its advantages, the phacoemulsification technique is associated with the high cost of purchasing and maintaining the necessary technology, which can be prohibitive in many low and middle-income countries^8^. MSICS is an alternative, but not all surgeons are trained to perform this surgery with sufficient skill^28,29^. Thus, there is a mismatch between techniques such as MSICS that are accessible in low-resource settings, where the need for cataract surgery is high, and the technology to enable surgeons to provide skilful care. There is little research on simulation-based training for MSICS, particularly on its effect on operating room performance^27,30^. This research gap is addressed by our study.

Our findings suggest a considerable magnitude of effect with VR simulation-based training for MSICS surgeons, despite being a pilot study whose findings must be replicated in a larger confirmatory phase III RCT. Reduction of incident total errors by nearly one-half among surgeons early in their learning curve, if confirmed, could translate into significant public health impact when the training curriculum is deployed at a large scale. In fact, the curriculum evaluated in this study was structured such that it can be reproduced across different settings. Our study shows proof of feasibility to deploy the curriculum with consistency at multiple academic institutions in India, which is a relevant setting to evaluate it given the public health burden of visual impairment and blindness due to cataract^31^.

Our study has limitations. Trainee and faculty surgeons at participating institutions were not masked to the trainees’ assignment to EG or CG. Such masking is not feasible to implement in our study as designed. While it may have resulted in some peer-to-peer learning from trainees assigned to EG, such learning should also be expected in CG. In addition, peer-to-peer learning is not likely to significantly influence performance of trainees in the operating room because the key element of learning in EG is simulation-based deliberate practice. Lack of masking of trainee and faculty surgeons may have affected outcome ascertainment in the operating room. However, the primary source of outcome ascertainment in our study was through independent video review by three experts who were masked to the surgeon performing the tunnel construction. The curriculum for trainees in CG was heterogeneous because of variation in practices and standards in the participating institutions. However, this heterogeneity only helps to reinforce the effectiveness of the HelpMeSee Eye Surgery Simulator-based training curriculum across different settings. Although trainees in EG completed their first 20 procedures over a shorter time period than those in CG (25 days vs. 51.4 days), this is not likely to affect validity of our findings because the duration between successive cases was not large enough to suspect degradation of skill among trainees in CG. Our study had a small sample size by design because there was no prior evidence from RCTs to inform sample size calculation. Our findings provide this information to design a subsequent confirmatory multicentre RCT. Finally, our study recruited trainees from multiple sites, but it was limited to three highly specialized tertiary eye care centres with highly competitive residency training programs. Therefore, the observed effect in the sample of trainees included in this study is not necessarily applicable to trainees across all training institutions. As is clear from Table 1, the trainees in both groups included residents and fellows across different years of training. This heterogeneity is an important limitation to the study. A sample composed only of PGY-1, for example, could possibly bring greater consistency to the results.

With the ongoing global COVID-19 pandemic, it is likely that elective surgeries, especially those assigned to trainees, will decline. A survey by Nair et al. revealed that 72.5% of the surveyed ophthalmologists were not seeing patients during the COVID-19 lockdown, with a near-total cessation of elective surgeries during the peak of the pandemic^32^. While it is expected that elective surgeries such as cataract will resume in the coming months, simulation training prior to actual surgery will benefit residents and patients alike^33^.

In conclusion, this RCT provides evidence that novice surgeons learning in a VR simulation-based training curriculum, using the HelpMeSee Eye Surgery Simulator, make fewer errors while performing tunnel construction in their first 20 procedures in the operating room compared with surgeons in a conventional training curriculum.
